# Recurrent Syncope as a Presentation of Pulmonary Embolism

**DOI:** 10.7759/cureus.6623

**Published:** 2020-01-10

**Authors:** Kulachanya Suwanwongse, Nehad Shabarek

**Affiliations:** 1 Internal Medicine, Lincoln Medical Center, New York City, USA

**Keywords:** syncope, pulmonary embolism, pe

## Abstract

The diagnosis of pulmonary embolism is challenging particularly when patients present with vague and/or non-specific symptoms and signs. Misdiagnosis of pulmonary embolism can lead to death or severe morbidity. We reported a case of a 60-year-old woman presented with recurrent syncope who later was diagnosed as submassive pulmonary embolism. This case report highlights the importance of early diagnosis and management of pulmonary embolism to prevent life-threatening sequels. Pulmonary embolism should be considered as a differential diagnosis of patients presenting at an emergency department with syncope.

## Introduction

Pulmonary embolism (PE) is accountable for more than 100,000 deaths in the United States annually [1]. PE presentation varies from asymptomatic with incidentally finding to sudden cardiac arrest. Early diagnosis and management of PE are important to prevent life-threatening sequels. Failure to diagnose PE leads to devastating complications, with up to 30% of untreated patients die [2]. Syncope, as a presentation of PE, has been considered as a challenging diagnosis [3]. We presented a case of a patient who presented with recurrent syncope and later was found to have PE, to increase awareness of clinicians in including PE as a possible cause of recurrent syncope. 

## Case presentation

A 60-year-old female presented to the emergency department after an episode of syncope while walking. She lost consciousness for a minute without convulsion or urinary incontinence. She reported palpitation, dizziness, mild chest discomfort, and shortness of breath before syncope. She had a past medical history of hypertension, type 2 diabetes mellitus, and osteoporosis. Her medications included sitagliptin, glimepiride, valsartan-hydrochlorothiazide, and raloxifene. She had multiple episodes of syncope (more than 10) accompanying with shortness of breath, mild chest discomfort, and palpitation in the past year. She was then admitted to another hospital and stated that all investigations including electrocardiogram (EKG), 24-hour telemetry, echocardiogram, and cardiac stress test were normal but did not have a chest computed tomography (CT).

**Figure 1:**
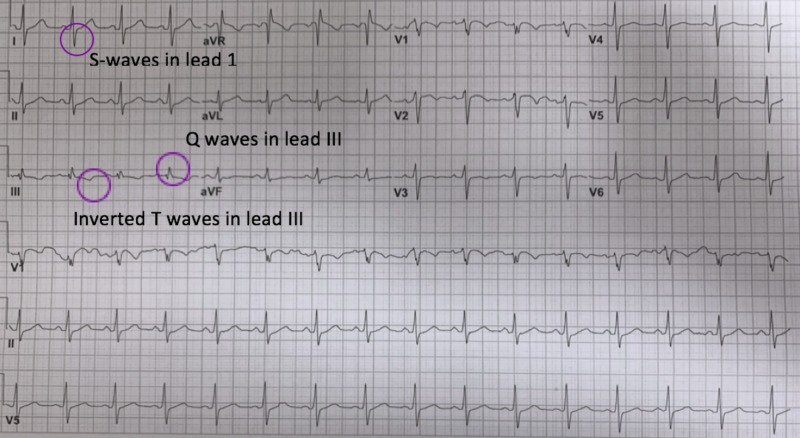
Electrocardiogram showed an S1Q3T3 pattern which includes S wave in lead I, Q wave in lead III with T-wave inversion

On initial evaluation, her blood pressure was 148/90 mmHg, heart rate was 93 beats per minute, oxygen saturation was 98% on room air and physical exam including heart and lungs and neurological exam were normal. Blood tests for glucose, electrolytes, creatinine, and complete blood counts were unremarkable except for mild anemia (Hb 10.9 g/dL) and mild elevation of troponin T of 0.015 ng/mL (normal <0.010 ng/mL). A head CT was normal. EKG was noted as normal sinus rhythms with non-specific ST and T wave changes but was reviewed later and found S1Q3T3 pattern, as demonstrated in Figure 1. She was advised to admit for further investigations but refused and signed out against medical advice. 

**Figure 2:**
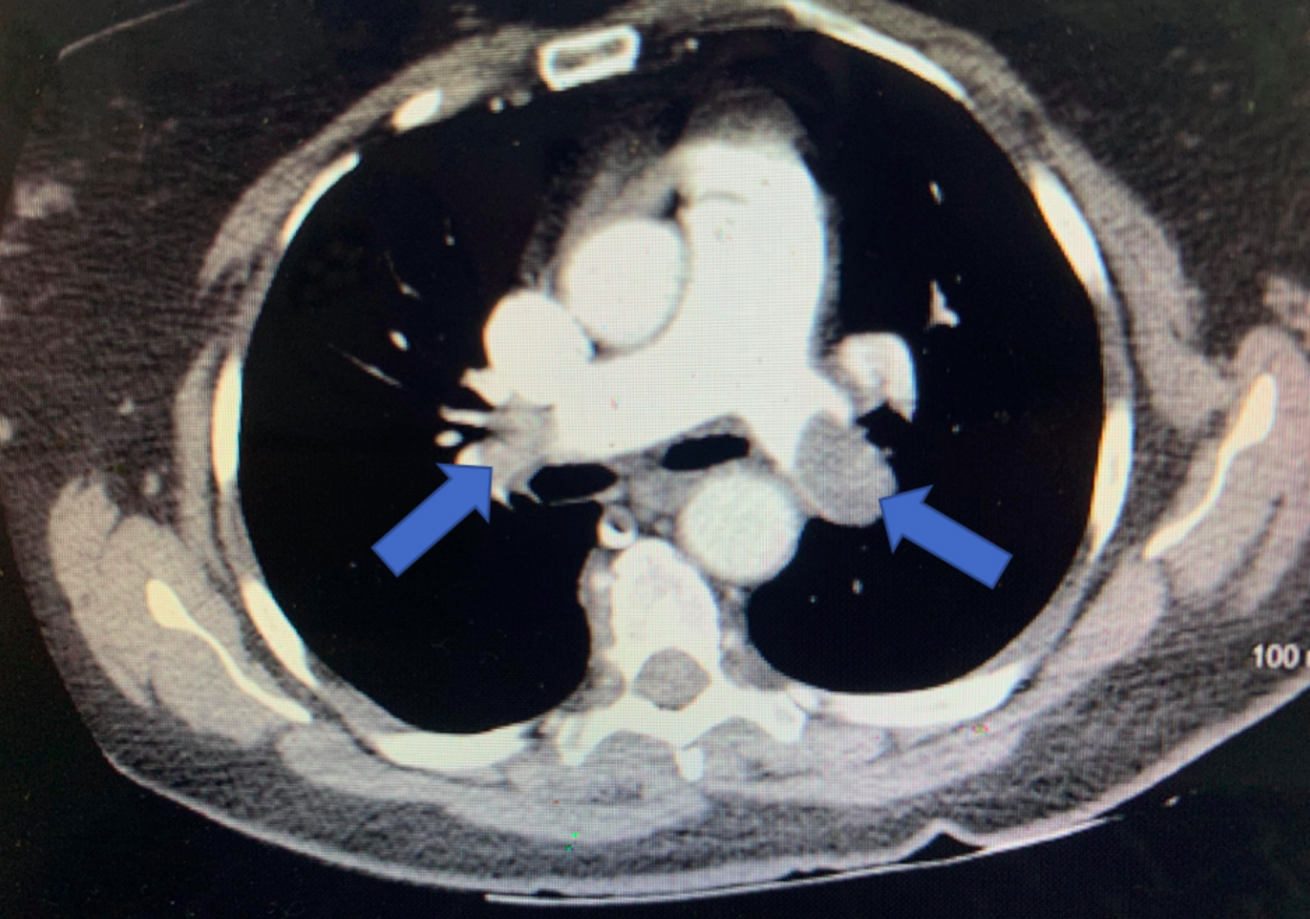
CT angiography showed prominent bilateral pulmonary emboli

While on her way out, she had another episode of syncope. She recovered spontaneously but reported worsening shortness of breath. On exam, she had tachypnea, a drop of blood pressure to 97/58 mmHg, heart rate was 91 beats per minute, and hypoxemia, with an oxygen saturation level of 60%. After resuscitation, a chest CT angiography was performed and showed prominent bilateral pulmonary emboli, as demonstrated in Figure 2. She was started on a heparin drip, transferred to the medical intensive care unit, and received catheter-directed thrombolysis. Doppler vascular sonography of bilateral lower extremities found occlusive intravenous thrombosis in the left popliteal vein with partially occlusive in distal left superficial femoral vein. She did not have any family history of deep vein thrombosis (DVT) but did have an increased risk for developing DVT from raloxifene. After a four-day course of hospital treatment, she was discharged on an oral anticoagulant (apixaban). Her shortness of breath was improved, and no recurrent syncope was reported after receiving anticoagulant.

## Discussion

PE is a differential diagnosis for syncope in most textbooks. However, when patients present with syncope, PE, a potentially fatal disease requiring urgent attention, is rarely considered [4]. This case is interesting as our patient had multiple episodes of syncope with extensive work-up, but without any suspicious of PE until developing severe hypoxemia. PE-induced syncope can be explained by three possible mechanisms. First, occlusion of more than 50% of pulmonary vessels leads to right ventricular failure and left ventricular filling impairment, causing a sudden drop in cardiac output and cerebral blood flow. Second, PE may induce arrhythmias from right ventricular strain. Third, the embolism itself may provoke a vasovagal reflex leading to neurogenic syncope [3,5].

Our case highlights the importance of thorough history taking and also illustrates a critical point in recognition of ‘S1Q3T3’ EKG pattern, which is rare but has high value in helping diagnosis of PE, especially a new onset ‘S1Q3T3’. The S1Q3T3 sign refers to a prominent S wave in lead I, Q wave, and inverted T wave in lead III, which reflects right ventricular strain. Any cause of cor pulmonale can result in an S1Q3T3 pattern on EKG, including PE, pneumothorax, and bronchospasm [6]. S1Q3T3 pattern has a sensitivity of 54% and a specificity of 62% in the diagnosis of PE [7]. Although S1Q3T3 is not specific nor sensitive, it is helpful when used with clinical contexts of patients in guiding the diagnosis of PE. 

## Conclusions

The diagnosis of PE in patients presenting with syncope is challenging. Physicians should have PE as a differential diagnosis for patients presented with syncope particularly with accompanying shortness of breath, respiratory distress, or hypoxemia. Early diagnosis and treatment of PE are critical to prevent morbidity and mortality from this condition.
